# A comparative study between 10-MHz and 15-MHz ultrasound probes for retinal evaluation in silicone-oil-filled globes

**DOI:** 10.1038/s41433-023-02464-5

**Published:** 2023-03-06

**Authors:** Maha A. Albadawi, Riham S. H. M. Allam, Noha M. M. Khalil, Iman M. Eissa

**Affiliations:** Department of Ophthalmology, Faculty of Medicine, Kasr Al Ainy School of Medicine, Cairo University, Cairo, Egypt

**Keywords:** Retinal diseases, Outcomes research, Diagnosis, Medical imaging

## Abstract

**Purpose:**

To compare 10-MHz and 15-MHz B-scan probes regarding the detection and localization of retinal detachment (RD) in silicone-oil-filled eyes.

**Methods:**

This cross-sectional observational study included 100 eyes (98 patients) -having media opacity precluding fundus examination- scheduled for silicone-oil removal. Patients were examined in the sitting-position using both frequencies one week preoperatively. Longitudinal and transverse scans were taken in primary-gaze, inferior, inferonasal, and inferotemporal positions to detect the presence/absence and extent of RD. Patients were sub-grouped according to axial lengths (AXLs), state of silicone emulsification, and globe filling. Agreement between sonographic and intraoperative observations was compared.

**Results:**

No statistically significant differences were found between 15-MHz and intra-operative findings regarding RD detection (*P* = 0.752) and accurate localization of inferior, inferonasal, and inferotemporal RD (*P* = 0.279, 0.606, and 0.599). There were statistically significant differences between 10-MHz and intra-operative findings regarding RD detection and localization (*P* < 0.001). The 15-MHz probe was superior to 10-MHz probe regarding the accuracy of RD detection and localization (94% and 47%, respectively). The accuracy of 15-MHz probe was 88%, 83%, and 85% in detecting and localizing inferior, inferonasal, and inferotemporal RD compared to 45%, 60%, and 62% with 10-MHz probe. The 15-MHz probe showed better sensitivity while 10-MHz probe showed better accuracy in eyes with short AXLs. The 10-MHz probe showed better sensitivity in patients with sonographic emulsification while15-MHz probe had better sensitivity in detecting vitreoretinal-interface disorders.

**Conclusion:**

The 15-MHz B-scan probe is more accurate in detecting and localizing recurrent RD in silicone-oil-filled globes with higher sensitivity in detecting vitreoretinal-interface disorders.

## Introduction

The use of silicone-oil in the treatment of retinal detachment (RD) has become widely used hence accurate sonographic evaluation of these eyes has become crucial. This is important in eyes having dense media opacities precluding fundus examination. The physical properties of silicone-oil led to attenuation and reflectivity of sound waves making interpretation of ultrasound (U/S) images in silicone-oil-filled eyes difficult [1].

U/S characteristics of eyes with silicone-oil tamponade include axial length (AXL) pseudo-elongation and an arc-shaped interface between the posterior global wall and the posterior edge of silicone bubble [2]. Few studies reported increased U/S accuracy by examining patients in the prone-position or by using Doppler-US [2, 3].

Others investigated the effect of using different U/S frequencies on accurate retinal evaluation and compared the 10-MHz and the 20-MHz B-scan probes [4]. Hewick, et al. found that the 20-MHz exhibited higher resolution images, was better in visualizing details of the posterior pole, and could differentiate layers such as the retina, choroid, and sclera [4, 5].

On the other hand, the 10-MHz probe had higher sensitivity and was better in visualizing low-intensity scatterers as vitreous opacities were difficult to be seen with higher frequencies and was preferred in silicone-oil-filled eyes as it avoids marked U/S attenuation that impedes evaluation using the 20-MHz probe [4].

The aim of this study is to compare the accuracy of the 10-MHz and the 15-MHz U/S probes in evaluating silicone-oil-filled globes regarding accuracy in detecting recurrent RD under silicone-oil, and to correlate B-scan findings with intra operative findings. To our knowledge, this study is the first to compare the 10-MHz and 15-MHz probes in imaging of silicone-oil-filled globes.

## Patients and methods

This comparative-observational-cross-sectional study was carried out during the period between April 2021 and October 2021. It included 100 eyes (98 patients) with silicone-oil-filled globes scheduled for silicone-oil removal. All patients were recruited from the retina outpatient clinics at Kasr Alainy hospital, Cairo University.

Data collection conformed to all local laws and was compliant with the tenets of the Declaration of Helsinki. The study protocol was approved by the research ethics committee of the faculty of medicine, at Cairo University (code: MD-257-2021). Written informed consent was obtained from all participants for the anonymous publication of their data.

The sample size was calculated using PASS-11 considering an alpha-error of 0.05, a confidence interval of 0.95, and a study power of 0.90. According to literature, the higher frequency probe had a superior resolution that better detects details at the posterior pole while the lower frequency probe had a superior sensitivity that can be used to examine low-intensity scatterers [4]. RD echo was localized between the echoes of silicone-oil and the global wall in 100% of the patients with clear optical media while only in 41.6% with non-transparent optical media [6].

Based on this data, a total sample size of 35 eyes was considered adequate to evaluate different U/S modes in detecting RD in silicone-oil-filled globes. We succeeded to recruit 100 silicone-oil-filled eyes candidates for silicone-oil removal who fulfilled the study inclusion criteria.

The study included patients of both genders who previously underwent pars-plana-vitrectomy with silicone-oil tamponade for rhegmatogenous RD, tractional RD, or for endophthalmitis. The study included phakic patients having dense cataract and pseudophakic/aphakic eyes having dense posterior capsule opacification, poorly dilatable pupils, and/or posterior synechia precluding adequate fundus examination.

Uncooperative and mentally ill patients and those having clear media were excluded from the study. Patients with any form of scleral buckles (encircling or segmental) were excluded from the study to avoid any distortion of the global wall that would affect the accuracy of B-scans. Patients with inverted-hypopyon ≥2 mm in height and gas-filled eyes were also excluded to avoid poor image quality that could affect examination results [7].

Patients meeting the inclusion criteria were subjected to full ophthalmological examination including assessment of the corrected visual acuity, intraocular pressure measurement using Goldmann-applanation-tonometry, and slit-lamp examination.

U/S was done using a contact B-scan probe coated with coupling-gel applied on closed eyelids. The patients were asked to open the other eye and to look in the required direction.

Examination was done in the sitting-position acquiring longitudinal and transverse scans (Supplemental information 1) in the primary-gaze, dead-inferior, inferonasal and inferotemporal positions, respectively. For each image, the investigator commented on the presence or absence of RD and the presence of any associated pathology. U/S was performed using a 15-MHz B-scan probe (Compact Touch, Quantel Medical, version 5.02, France) followed 1–2 days later by the 10-MHz B-scan probe (Sonomed EZ Scan AB5500 and Scanner).

The sitting-position has been chosen to allow silicone-oil to float [8, 9] and to get images of the double-globe (the large globe is due to the presence of silicone pseudo-elongation, and the small globe devoid of silicone). Thus, the presence of the small globe gives us the opportunity to study the inferior retina and to get the benefit of the higher resolution of the 15-MHz B-scan probe [10] avoiding the disadvantage of increased attenuation of sound waves inside silicone-oil.

U/S examination was performed by one examiner for each procedure (examiner-1 for the 10-MHz and examiner-2 for the 15-MHz), who were both masked to the clinical data and to each other’s results. The two investigators have the same efficiency (an inter-observer agreement has been performed prior to the initiation of this study on 15 patients and revealed a significant good inter-observer agreement “κ = 0.65, *P* = 0.001”).

All examination settings-except for the gain and time gain compensation (TGC)-were identical when comparing the 10-MHz and 15-MHz images. The decibel (dB) gain was adjusted when using each probe to give the optimal images (range from 90–105 dB). We started the examination using the highest gain of the machine (110 dB in the 15 MHz and 100 dB in the 10 MHz probes) then the gain was re-adjusted to confirm the presence of retinal detachment avoiding echoes induced by other membranes at the interface that would disappear by a slight lowering of the gain. Concomitantly, TGC was adjusted manually to obtain the clearest view for the posterior global wall and avoid artifacts and silicone-induced signal attenuation [11, 12] (having maximum values of 30 dB/cm in the 15 MHz and 25.5 dB/cm in the 10 MHz probes).

Patients were divided into the following sub-groups:

- Myopes, emmetropes, and hyperopes; according to their AXL measured by A-scan after changing machine settings according to the type of silicone used as per patients’ records and the lens status. A-scan was performed using an 11-MHz A-scan probe (Compact Touch, Quantel Medical, version 5.02, France). These were the measurements used in statistical correlations.
  **N.B:** For AXL measurements taken from the 15 & 10 MHz B & A-scans, a correction factor was applied for calculation (0.64 for 1000cst and 0.68 for 5000cst silicone oil) after excluding the spikes of the close eye lid to the anterior cornea [13–15].
- Patients with emulsified or non-emulsified silicone.

The pre-operative conditions were compared with subsequent observations during surgery for silicone-oil removal to investigate agreement between preoperative ultrasonic findings using both frequencies and intra-operative findings (the intra-operative observer was the same for all study cases and was responsible for attending all the surgeries and documenting the retinal status in all cases). This would give feedback on the reliability of each probe in the preoperative detection of possible recurrent RD. The time interval between both U/S scans and the surgery was less than 1 week.

## Statistical analysis

Data were analysed using Statistical Package for Social Sciences (SPSS) version 22. Data were expressed as mean, standard deviation, and range for quantitative data. Categorical data were summarized as frequency (count) and relative-frequency (percentage). Quantitative variables were compared using the paired-samples *t*-test. Chi-square (χ2) test was performed to compare categorical data. An exact test was used when the expected frequency was <5. Cohen’s-kappa-coefficient (κ) was used to express inter-groups agreement. Cross-tabulations were run; accuracy, positive-predictive-value (PPV), negative-predictive-value (NPV), and sensitivity/specificity percentages were calculated taking the intra-operative findings as reference. The confidence interval was set to 95% with a 5% margin of error. *P* ≤ 0.05 was considered significant.

## Results

A total of 100 eyes (98 patients) were included in this study with a male: female ratio of 2.125:1 and a mean age of 40.98 ± 17.67 years. Prior to surgical intervention, 12% of the study participants had emulsified silicone in the anterior chamber as detected on gonioscopy or as minimal inverted hypopyon, 31% were phakic having dense cataract, 57% were pseudophakic with dense posterior capsular opacification and 12% were aphakic with poorly dilatable pupils and/or posterior synechia. The demographic and clinical data of the study subjects are summarized in Table 1.

**Table 1:** The demographic and clinical data of the study subjects.

Clinical factors		
Age (years)	Mean ± SD	40.98 ± 17.67
	Range	5–68
Sex (Count, %)	Females	32 (32%)
	Males	68 (68%)
Visual Acuity (Count, %)	≤1/60	64 (64%)
	>1/60 - ≤3/60	17 (17%)
	>3/60 - ≤6/60	8 (8%)
	>6/60	11 (11%)
Duration of silicone-oil filling (months)	Mean ± SD	9.84 ± 7.96
	Range	2–48
Laterality (Count, %)	Right eye	58 (58%)
	Left eye	42 (42%)
Bilateral cases (Count, %)	Bilateral	2 (4%)
	Unilateral	96 (96%)
Lens status (Count, %)	Phakic	31 (31%)
	Pseudophakic	57 (57%)
	Aphakic	12 (12%)
IOP^*^ (mmHg)	Mean ± SD	13.6 ± 7
	Range	2–40
Preoperative treatment (Count, %)	No treatment	80 (80%)
	Anti-glaucoma topical treatment	18 (18%)
	Topical Steroids	2 (2%)
Indications for pars plana vitrectomy (Count, %)	Rhegmatogenous retinal detachment	82 (82%)
	Tractional retinal detachment	9 (9%)
	Endophthalmitis	8 (8%)
	Vitreous haemorrhage	1 (1%)
Emulsified silicone in the anterior chamber (Count, %)	Yes	12 (12%)
	No	82 (82%)

IOP^*^ Intraocular pressure

Comparing the results of the 10-MHz & 15-MHz B-scans to each other as-well-as to the intra-operative results, there was no significant difference between the 15-MHz B-scan and the intra-operative findings as illustrated in Appendix 1. Further post-hoc analyses showed significant differences between the 10-MHz B-scan and the intra-operative findings; as summarized in Appendix 1; as-well-as between the 10-MHz and the 15-MHz B-scan findings as illustrated in Table 2.

**Table 2:** Comparison between the 15-MHz B-scan and the 10-MHz B-scan regarding detection and localization of RD* under silicone.

		15-MHz B-scan	10-MHz B-scan	*P*-value
		No. (%)	No. (%)	
General impression (Presence or absence of RD* regardless the location)	Yes	29 (29%)	74 (74%)	<0.001**
	No	71(71%)	26 (26%)	
Primary position	Yes	10 (10%)	21 (21%)	0.032**
	No	90 (90%)	79 (79%)	
Dead inferior	Yes	16 (16%)	57 (57%)	<0.001**
	No	84 (84%)	43 (43%)	
Infero-nasal	Yes	20 (20%)	49 (49%)	<0.001**
	No	80 (80%)	51 (51%)	
Infero-temporal	Yes	19 (19%)	48 (48%)	<0.001**
	No	81 (81%)	52 (52%)	

^*^RD Retinal detachment.

^**^P-values ≤ 0.05 are considered significant.

### Sensitivity, specificity, and accuracy of 10-MHz & 15-MHz B-scans regarding detection and accurate localization of RD under silicone

The 15-MHz B-scan was superior to the 10-MHz B-scan regarding its accuracy in RD detection (94% compared to 47% respectively) as-well-as its accurate localization. Accuracy of the 15-MHz B-scan probe was 88%, 83%, and 85% in localizing inferior, infero-nasal, and infero-temporal RD respectively compared to 45%, 60%, and 62% with the 10-MHz B-scan.

Also, the 15-MHz B-scan was superior to the 10-MHz B-scan in localizing inferior RD with higher sensitivity, specificity, PPV, NPV, and accuracy.

A comparison between both frequencies regarding the detection and localization of RD at all locations is shown in Table 3 and Supplemental information 2–4.

**Table 3:** Sensitivity, specificity, PPV^±^, NPV^Δ^, and accuracy in RD^*^ detection by the 15-MHz versus 10-MHz B-scan probes.

		Sensitivity	Specificity	PPV^±^	NPV^Δ^	Accuracy
General impression about the presence or absence of RD* regardless of the location	15-MHz B-scan	77.8%	89.0%	72.4%	91.5%	94%
	10-MHz B-scan	88.9%	31.5%	32.4%	88.5%	47%
Dead inferior	15-MHz B-scan	59.1%	96.2%	81.3%	89.3%	88%
	10-MHz B-scan	54.5%	42.3%	21.1%	76.7%	45%
Inferonasal	15-MHz B-scan	56.5%	90.9%	65%	87.5%	83%
	10-MHz B-scan	69.6%	57.1%	32.7%	86.3%	60%
Inferotemporal	15-MHz B-scan	59.1%	92.3%	68.4%	88.9%	85%
	10-MHz B-scan	72.7%	59.0%	33.3%	88.5%	62%

^*^RD Retinal detachment

^±^*PPV* Positive-predictive-value (Percentage of true positive cases compared to the whole positive of the same test).

^Δ^*NPV* Negative-predictive-value (Percentage of true negative cases compared to the whole negative of the same test).

### Agreement between each frequency of B-scan and intra-operative findings

The 15-MHz B-scan and the intra-operative findings showed good agreement regarding localization of inferior RD “κ = 0.612, *P* < 0.001”, moderate agreement regarding localization of infero-nasal and infero-temporal RD “κ = 0.497, *P* < 0.001” and “κ = 0.540, *P* < 0.001” respectively and poor agreement in detecting RD regardless its location “κ = 0.143, *P* < 0.001”.

The 10-MHz B-scan and the intra-operative findings showed poor agreement regarding RD detection as-well-as its localization. The agreement coefficient was “κ = 0.132, *P* = 0.039”, “κ = −0.020, *P* = 0.792”, “κ = zero” and “κ = zero” regarding detection of RD regardless the location, inferior, inferonasal and inferotemporal RD respectively.

The 10-MHz B-scan and the 15-MHz B-scans showed poor agreement regarding RD detection as-well-as its localization. The agreement coefficient was “κ = 0.045, *P* = 0.075”, “κ = 0.067, *P* = 0.461”, “κ = −0.041, *P* = 0.537”, “κ = zero” and “κ = zero” regarding detection of RD regardless the location, 1ry position, inferior, inferonasal and inferotemporal RD, respectively.

### The state of silicone (emulsification and filling)

Sonographic emulsification was detected in 90% of the study subjects using both B-scans while intra-operatively; it was detected in 77% of them (*P* = 0.010).

A significant agreement was found between the 15-MHz B-scan and the intra-operative findings, between the 10-MHz B-scan and the intra-operative findings as-well-as between the 15-MHz and the 10-MHz B-scans regarding the detection of silicone emulsification (87%, 83% and 94%) respectively.

Comparing both B-scans in the whole study participants to those with sonographic emulsification (90 subjects), the 10-MHz B-scan showed improvement in sensitivity (from 88.9% to 91.7%) and NPV (from 88.5% to 90.9%). However, the 15-MHz B-scan showed no improvements as shown in Appendix 2.

Comparing both B-scans in the whole study group to those with incomplete silicone filling (74 subjects), the 10-MHz B-scan showed improvement in sensitivity (from 88.9% to 91.3), PPV (from 32.4% to 37.5%) and accuracy (from 47% to 50%). However, the 15-MHz B-scan showed improvement regarding sensitivity (from 77.8% to 87%) and PPV (from 72.4% to 93.5%) as shown in Appendix 2.

### AXL measurement

The mean AXL for all study participants was measured using both 15-MHz and 10-MHz B-scans as-well-as the 11-MHz A-scan probe. The resultant values were 24.51 ± 2.57 mm (range 20–33.33 mm), 23.69 ± 2.48 mm (range 20–33 mm), and 25.09 ± 3.16 mm (range 19.17–35.6 mm) respectively. Comparing the results of the 3 probes, they showed a significant difference (*P* = 0.002).

Further comparisons showed that the highest difference in AXL was found between the 10-MHz B-scan and the A-scan (*P* = 0.001) while no significant differences were found between the A-scan and the 15-MHz B-scan (*P* = 0.307) as-well-as between the 10-MHz and 15-MHz B-scans (*P* = 0.090).

28.57% of the A-scans readings were taken by the manual-mode compared to 71.42% by the Auto-mode where the lens status and silicone-oil viscosity (in centistokes, cst) have been selected. 92 of the study subjects had silicone 5000 cst while 6 had silicone 1000 cst. According to the A-scan; 52 of our study subjects were myopes with AXLs>24 mm, 13 subjects were hypermetropes with AXLs<22 mm and 33 subjects were emmetropes with AXLs ranging between 22–24 mm. We failed to do A-scan for 2 of our participants due to nystagmus and poor fixation in the primary position.

Further analysis of the effect of AXL on the accuracy of B-scan showed that in axial myopia (52 eyes), both the 15-MHz and the 10-MHz B-scans showed a reduction in Sensitivity, PPV, and accuracy. On the contrary, in axial hypermetropia (13 eyes), the 15-MHz B-scan showed improvement in sensitivity and the 10-MHz B-scan showed improvement in specificity, PPV, and accuracy.

On the other hand, in axial emmetropia (33 eyes) the 15-MHz B-scan showed improvement in sensitivity, specificity and in PPV and the 10-MHz B-scan showed improvement in sensitivity and in PPV.

To summarize the effect of AXL on B-scans, as the AXL decreases the sensitivity of the 15-MHz B-scan probe and the accuracy and PPV of the 10-MHz B-scan increase. Data are summarized in Appendix 3.

### Vitreoretinal-interface findings

**Fig. 1:**
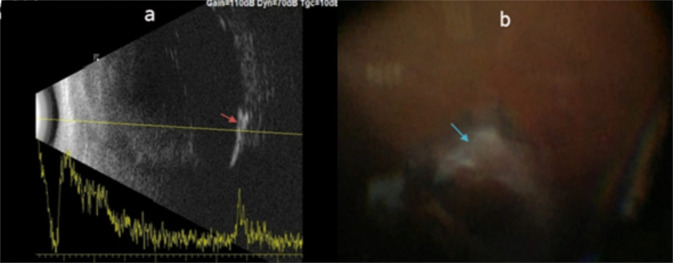
Comparing the 15-MHz B-scan to the intraoperative findings regarding detection of vitreoretinal-interface disorders. **a** Inferior chorioretinal degenerations and scaring as detected by the 15 MHz B-scan probe (red arrow), **b** An intra-operative photo showing an inferior chorioretinal scar (blue arrow).

The 15-MHz and 10-MHz B-scans were compared to each other as-well-as to the intra-operative findings regarding the detection of vitreoretinal-interface disorders; (proliferative vitreo-retinopathy [PVR], chorio-retinal scars [CRS], epiretinal membranes [ERM], Vitreous membranes, vitreo-retinal traction [VRT] and retinectomy edge). There were no significant differences between the 15-MHz B-scan and the intra-operative findings apart from the detection of CRS and ERM (*P* = 0.001 for both). The results are further illustrated in Appendix 4 and Fig. 1.

The 10-MHz B-scan could only detect vitreous membranes (one eye compared to 5 eyes intra-operatively) and retinectomy edges (9 eyes compared to 6 eyes intra-operatively) with no significant differences when compared to the intra-operative findings (*P* = 0.097 and 0.421), respectively.

Comparing the 10-MHz and the 15-MHz B-scans regarding the detection of vitreous membranes (6 eyes with the 15-MHz B-scan probe and one eye with the 10-MHz B-scan probe) and retinectomy edges (9 eyes with both B-scan frequencies); it showed no significant differences (*P* = 0.054 and 1.000), respectively.

### Sensitivity and specificity of the 10-MHz & 15-MHz B-scans regarding detection of vitreoretinal-interface findings

The 15-MHz B-scan was sensitive in detecting ERM, VRT, Vitreous membranes, CRS, PVR, and retinectomy edges by 60%, 50%, 40%, 32.7%, 20%, and 16.7% respectively. While its specificity in their detection was 51.6%, 98%, 95.8%, 88.2%, 92.6% and 91.5% respectively. The 10-MHz B-scan showed equal sensitivity with the 15-MHz B-scan regarding the detection of retinectomy edges (16.7%) and zero sensitivity in detecting vitreous membranes. Moreover, the 10 MHz B-scan showed the specificity of 98.9% and 91.5 regarding the detection of vitreous membranes and retinectomy edges respectively.

## Discussion

The results of the current study demonstrate that the 15-MHz B-scan is superior to the 10-MHz B-scan regarding its accuracy in RD detection (94% with the 15-MHz compared to 47% with the 10-MHz) as-well-as its accurate localization with higher specificity, PPV, and NPV.

On the other hand, the 10-MHz B-scan showed more sensitivity in RD detection (88.9% compared to 77.8% with the 15-MHz). This is noticeable in eyes with near-complete silicone filling which hampered good visualization of the small globe devoid of silicone. In these eyes, the 10-MHz was better due to the lower attenuation of sound waves that allow better retinal evaluation at the silicone-retina interface.

In the current study, there was a large difference regarding the specificity, PPV, NPV, and accuracy in RD detection where the 15-MHz was superior to the 10-MHz.This gap in specificity (89% for the 15-MHz, 31.5% for the 10-MHz) was probably attributed to the higher false-positive results found with the 10-MHz probe.

In a study done on 20 patients with recurrent RD under silicone; the US was able to detect RD in only 41.6% of the cases. However, the authors did not mention the frequency used [6]. This was different from our study where we found a higher sensitivity with the 10-MHz versus the 15-MHz (88.9% and 77.8%respectively).

In the present study, AXL has been measured using both B-scans in comparison to the 11-MHz A-scan. A significant difference has been found between the 3 probes with the highest difference between the 10-MHz B-scan and the 11-MHz A-scan while no significant difference has been found between the 15-MHz B-scan and the 11-MHz A-scan. This result means that the 10-MHz B-scan could be less accurate in AXL measurement.

In this work, it has been found that as the AXL decreases, the sensitivity of the 15-MHz B-scan increases from 75% in myopes to 81.8% in emmetropes and 83.3% in hyperopes. A similar effect was noticed with the 10-MHz B-scan which showed improvement in accuracy and PPV as the AXL decreases. These findings could be explained by the lower attenuation of the sound waves as the AXL decreases in silicone-oil-filled eyes, which relatively increases the resolution of the B-scan images on the posterior global wall.

The current study compared both B-scans with the intra-operative findings regarding the detection of silicone emulsification; there was a significant difference between them with the 15-MHz probe showing a higher level of agreement and the findings were not affected by silicone state. The 10-MHz B-scan showed improvement in sensitivity and NPV probably attributed to the higher penetration of sound waves in presence of silicone emulsification which allows better visualization of the silicone retina interface.

Comparing both B-scans of the whole study group to those with incomplete silicone filling, the 10-MHz B-scan showed improvement in sensitivity, PPV, and accuracy while the 15-MHz B-scan showed improvement in sensitivity and PPV. The reported improvements are probably attributed to the presence of a larger area in the inferior retina devoid of silicone in eyes with incomplete silicone filling, which gives the appearance of the double-globe on B-scan imaging; the small globe allows better visualization and evaluation of the inferior retina.

In the present study, both B-scans were compared regarding their sensitivity in detecting vitreoretinal-interface disorders. There were no significant differences between the 15-MHz B-scan and the intra-operative findings apart from the detection of CRS and ERM (*P* < 0.001 for both). However, the 10-MHz B-scan failed to detect PVR, CRS, ERM, and VRT. The higher sensitivity of the 15-MHz B-scan could be attributed to the higher resolution and hence better visualization of the inferior vitreoretinal-interface in eyes with incomplete silicone.

An agreement has been measured between the 15-MHz B-scan and the intra-operative findings and it showed good agreement regarding localization of inferior RD (κ = 0.612), moderate agreement regarding localization of infero-nasal and infero-temporal RD (κ = 0.497 and 0.540).

On the other hand, the 10-MHz B-scan showed poor agreements regarding RD detection as-well-as its localization in comparison to both the 15-MHz B-scan and the intra-operative findings.

To our knowledge, this is the first work comparing the 10-MHz and the 15-MHz B-scans in imaging silicone-oil-filled globes having media opacity and further correlating these findings with the intra-operative findings. However, the current study may have the limitation of the presence of a human factor; the sonographic assessment was done by 2 different investigators who were masked from each other’s results.

The strength of the current study may lie in the large sample size (100 silicone-filled-eyes) and the measures taken to decrease subjectivity (the inter-observer agreement performed prior to initiation of the study, masking between observers’ results, B-scans done less than one week prior to surgery, and the intra-operative observer being the same for all study cases).

Our study concludes that the 15-MHz B-scan is more accurate in detecting and localizing recurrent RD under silicone. As the AXL decreases, the sensitivity of the 15-MHz B-scan as-well-as the accuracy and the PPV of the 10-MHz B-scan showed improvement. Sonographic emulsification improves the sensitivity and NPV of the 10-MHz B-scan. Both probes showed improvement in sensitivity and PPV in patients with incomplete silicone filling. The 15-MHz probe also showed higher sensitivity in detecting vitreoretinal-interface disorders in silicone-oil-filled eyes.

We recommend using the 15-MHz B-scan pre-operatively in imaging silicone-oil-filled globes with media opacity. The examination is better done in the sitting-position to allow visualization of the double-globe sign and to get the benefit of the higher resolution of the 15-MHz B-scan in visualizing the inferior retina.

## Summary

### What was known before

- B-scan imaging is essential for retinal evaluation in eyes with media opacity; however, the interpretation of these images is difficult due to the ultrasound properties of silicone oil.
- Trials to increase the accuracy of B-scan imaging in these eyes were done by applying the sitting or prone position or by using Doppler ultrasonography
- The 10 MHz probe was preferred in eyes filled with silicone oil (because high attenuation makes scanning with the 20 MHz probe impossible).

### What this study adds

- This is the 1st study to compare 10 MHz and 15 MHz B-scan probes in imaging silicone-oil-filled globes.
- The 15 MHz B-scan probe is more accurate than the 10 MHz B-scan in the retinal evaluation and more sensitive in detecting vitreoretinal interface disorders in the eye with incomplete silicone filling (because of the higher resolution of the 15 MHz probe in imaging the inferior retina which is devoid of silicone; double-globe sign).
- The sitting-position is evaluated in large sample size and is preferred in imaging silicone-oil-filled globe for better evaluation of the inferior retina which is the commonest site of recurrent RD in these eyes.

### Supplementary information

Appendix 1

Appendix 2

Appendix 3

Appendix 4

SDC 1

SDC 2

SDC 3:

SDC 4:

Supplemental information

## Data Availability

The datasets used during the current study are available from the corresponding author upon request.

## References

[1] Mundt GH, Hughes WF (1956). Ultrasonics in ocular diagnosis. Am J Ophthalmol.

[2] Li YF, Li DJ, Wang ZY, Chen W, Zhao Q, Cui R (2017). Ultrasonic diagnosis of retinal detachment in eyes with silicone oil tamponade. Chin J Ophthalmol.

[3] Kumar A, Sharma N, Singh R (1998). Prone position ultrasonography in silicone-filled eyes. Acta Ophthalmol Scand.

[4] Hewick SA, Fairhead AC, Culy JC, Atta HR (2004). A comparison of 10 MHz and 20 MHz ultrasound probes in imaging the eye and orbit. Br J Ophthalmol.

[5] Agarwal A, Maleki A, Nguyen QD. History and principles of ocular ultrasonography. In Clinical Atlas of Ophthalmic Ultrasound 2019 (pp. 1–6). Springer, Cham.

[6] Krásnik V, Strmen P, Furdová A, Hasa J (1998). Ultrazvuková diagnostika amócie sietnice po vnútornej tamponáde silikónovým olejom (Ultrasonic diagnosis of retinal detachment after internal tamponade with silicone oil). Cesk Slov Oftalmol.

[7] Park SH, Lee SJ (2007). The results of B-scan ultrasonography in different positions after vitrectomy and gas tamponade. Korean J Ophthalmol.

[8] Barca F, Caporossi T, Rizzo S (2014). Silicone oil: different physical proprieties and clinical applications. BioMed Res Int.

[9] Mondelo‐García C, Bandín‐Vilar E, García‐Quintanilla L, Castro‐Balado A, Del Amo EM, Gil‐Martínez M (2021). Current situation and challenges in vitreous substitutes. Macromol Biosci.

[10] Cha JH, Chang JH (2014). Development of 15 MHz 2-2 piezo-composite ultrasound linear array transducers for ophthalmic imaging. Sens Actuators A: Phys.

[11] Mohamed IE, Mohamed MA, Yousef M, Mahmoud MZ, Alonazi B (2018). Use of ophthalmic B-scan ultrasonography in determining the causes of low vision in patients with diabetic retinopathy. Eur J Radio Open.

[12] Khazaei H, Khazaei D, Ashraf D, Mikkilineni S, Ng JD (2022). Overview of orbital. Ultrasonography Ann Ophthalmol Vis Sci.

[13] Takei K, Sekine Y, Okamoto F, Hommura S (2002). Measurement of axial length of eyes with incomplete filling of silicone oil in the vitreous cavity using x-ray computed tomography. Br J Ophthalmol.

[14] Larkin GB, Flaxel CJ, Leaver PK (1998). Phacoemulsification and silicone oil removal through a single corneal incision. Ophthalmology.

[15] Murray DC, Potamitis T, Good P, Kirkby GR, Benson MT (1999). Biometry of the silicone oil-filled eye. Eye.

