# Supplementary material for: A comparative study between 10-MHz and 15-MHz ultrasound probes for retinal evaluation in silicone-oil-filled globes
Source: Eye (Lond). 2023 Mar 6;37(14):3020–5. doi: 10.1038/s41433-023-02464-5 (PMC10516990; doi:10.1038/s41433-023-02464-5)
Supplement: Supplementary file 1 — Appendix 1 [file 41433_2023_2464_MOESM1_ESM.docx]

**Appendix (1): Comparison between the 15-MHz B-scan, the 10-MHz B-scan and the intra-operative findings regarding detection and localization of RD* under silicone**

|  | | 15-MHz B-scan | Intra-operative | P-value | 10-MHz B-scan | Intra-operative | P-value |
| --- | --- | --- | --- | --- | --- | --- | --- |
|  |  | No. (%) | No. (%) |  | No. (%) | No. (%) |  |
| General impression (presence or absence of RD* regardless the location) | Yes | 29 (29%) | 27 (27%) | 0.752 | 74 (74%) | 27 (27%) | < 0.001** |
|  | No | 71 (71%) | 73 (73%) |  | 26 (26%) | 73 (73%) |  |
| Dead inferior | Yes | 16 (16%) | 22 (22%) | 0.279 | 57 (57%) | 22 (22%) | < 0.001** |
|  | No | 84 (84%) | 78 (78%) |  | 43 (43%) | 78 (78%) |  |
| Infero-nasal | Yes | 20 (20%) | 23 (23%) | 0.606 | 49 (49%) | 23 (23%) | <0.001** |
|  | No | 80 (80%) | 77 (23%) |  | 74 (74%) | 27 (27%) |  |
| Infero-temporal | Yes | 19 (19%) | 22 (22%) | 0.599 | 26 (26%) | 73 (73%) | < 0.001** |
|  | No | 81 (81%) | 78 (78%) |  | 57 (57%) | 22 (22%) |  |

**RD: Retinal detachment*

***P-values ≤ 0.05 are considered significant*
