# Supplementary material for: A comparative study between 10-MHz and 15-MHz ultrasound probes for retinal evaluation in silicone-oil-filled globes
Source: Eye (Lond). 2023 Mar 6;37(14):3020–5. doi: 10.1038/s41433-023-02464-5 (PMC10516990; doi:10.1038/s41433-023-02464-5)
Supplement: Supplementary file 2 — Appendix 2 [file 41433_2023_2464_MOESM2_ESM.docx]

**Appendix (2): Sensitivity, specificity, PPV^±^, NPV^Δ^ and accuracy regarding RD* detection in study subjects with sonographic emulsification and incomplete silicone filling using the 15 and 10-MHz B-scans in comparison to the whole study group**

|  |  | Sensitivity | Specificity | PPV^±^ | NPV^Δ^ | Accuracy |
| --- | --- | --- | --- | --- | --- | --- |
| Whole group | 15-MHz  B-scan | 77.8% | 89.0% | 72.4% | 91.5% | 94% |
|  | 10-MHz  B-scan | 88.9% | 31.5% | 32.4% | 88.5% | 47% |
| Emulsified silicone | 15-MHz  B-scan | 76.9% | 87.5% | 71.4% | 90.3% | 84.44% |
|  | 10-MHz  B-scan | 91.7% | 30.3% | 32.4% | 90.9% | 46.67% |
| Incomplete silicone filling | 15-MHz  B-scan | 87.0% | 84.3% | 93.5% | 71.4% | 85.13% |
|  | 10-MHz B-scan | 91.3% | 31.4% | 37.5% | 88.9% | 50% |

***RD: Retinal detachment**

**^±^PPV: Positive-predictive-value (Percentage of true positive cases compared to the whole positive of the same test)**

**^Δ^NPV: Negative-predictive-value (Percentage of true negative cases compared to the whole negative of the same test)**
