# Supplementary material for: A comparative study between 10-MHz and 15-MHz ultrasound probes for retinal evaluation in silicone-oil-filled globes
Source: Eye (Lond). 2023 Mar 6;37(14):3020–5. doi: 10.1038/s41433-023-02464-5 (PMC10516990; doi:10.1038/s41433-023-02464-5)
Supplement: Supplementary file 3 — Appendix 3 [file 41433_2023_2464_MOESM3_ESM.docx]

**Appendix (3): Sensitivity, specificity, PPV^±^, NPV^Δ^ and accuracy in RD* detection in myopic, hypermetropic and emmetropic patients using the 15-MHz and 10-MHz B-scans in comparison to the whole study group**

|  |  | Sensitivity | Specificity | PPV^±^ | NPV^Δ^ | Accuracy |
| --- | --- | --- | --- | --- | --- | --- |
| Whole group | 15-MHz B-scan | 77.8% | 89.0% | 72.4% | 91.5% | 94% |
|  | 10-MHz B-scan | 88.9% | 31.5% | 32.4% | 88.5% | 47% |
| Myopes | 15-MHz B-scan | 75.0% | 93.2% | 66.7% | 95.3% | 90.38% |
|  | 10-MHz B-scan | 87.5% | 34.1% | 19.4% | 93.8% | 42.3% |
| Hypermetropes | 15-MHz B-scan | 83.3% | 57.1% | 62.5% | 80.0% | 69.23% |
|  | 10-MHz B-scan | 83.3% | 42.9% | 55.6% | 75.0% | 61.54% |
| Emmetropes | 15-MHz B-scan | 81.8% | 90.9% | 81.8% | 90.9% | 87.88% |
|  | 10-MHz B-scan | 90.9% | 22.7% | 37% | 83.3% | 45.45% |

***RD: Retinal detachment**

**^±^PPV: Positive-predictive-value (Percentage of true positive cases compared to the whole positive of the same test)**

**^Δ^NPV: Negative-predictive-value (Percentage of true negative cases compared to the whole negative of the same test)**
