# Supplementary material for: A comparative study between 10-MHz and 15-MHz ultrasound probes for retinal evaluation in silicone-oil-filled globes
Source: Eye (Lond). 2023 Mar 6;37(14):3020–5. doi: 10.1038/s41433-023-02464-5 (PMC10516990; doi:10.1038/s41433-023-02464-5)
Supplement: Supplementary file 4 — Appendix 4 [file 41433_2023_2464_MOESM4_ESM.docx]

**Appendix (4): Comparison between the 15-MHz B-scan probe and the intra-operative regarding detection of vitreoretinal interface findings**

|  | | 15-MHz B-scan | Intra-operative | P-value |
| --- | --- | --- | --- | --- |
|  |  | No. (%) | No. (%) |  |
| PVR | Yes | 8 (8%) | 5 (5%) | 0.390 |
|  | No | 92 (92%) | 95 (95%) |  |
| CRS | Yes | 22 (22%) | 49 (49%) | <0.001* |
|  | No | 78 (78%) | 51 (51%) |  |
| ERM | Yes | 49 (49%) | 5 (5%) | <0.001* |
|  | No | 51 (51%) | 95 (95%) |  |
| Vitreous membranes | Yes | 6 (6%) | 5 (5%) | 0.756 |
|  | No | 94 (94%) | 95 (95%) |  |
| VRT | Yes | 3 (3%) | 2 (2%) | 0.651 |
|  | No | 97 (97%) | 98 (98%) |  |
| Retinectomy | Yes | 9 (9%) | 6 (6%) | 0.421 |
|  | No | 91 (91%) | 94 (94%) |  |

****P-values* ≤0.05 are considered significant**
