# Supplementary figures and images for: A comparative study between 10-MHz and 15-MHz ultrasound probes for retinal evaluation in silicone-oil-filled globes
Source: Eye (Lond). 2023 Mar 6;37(14):3020–5. doi: 10.1038/s41433-023-02464-5 (PMC10516990; doi:10.1038/s41433-023-02464-5)

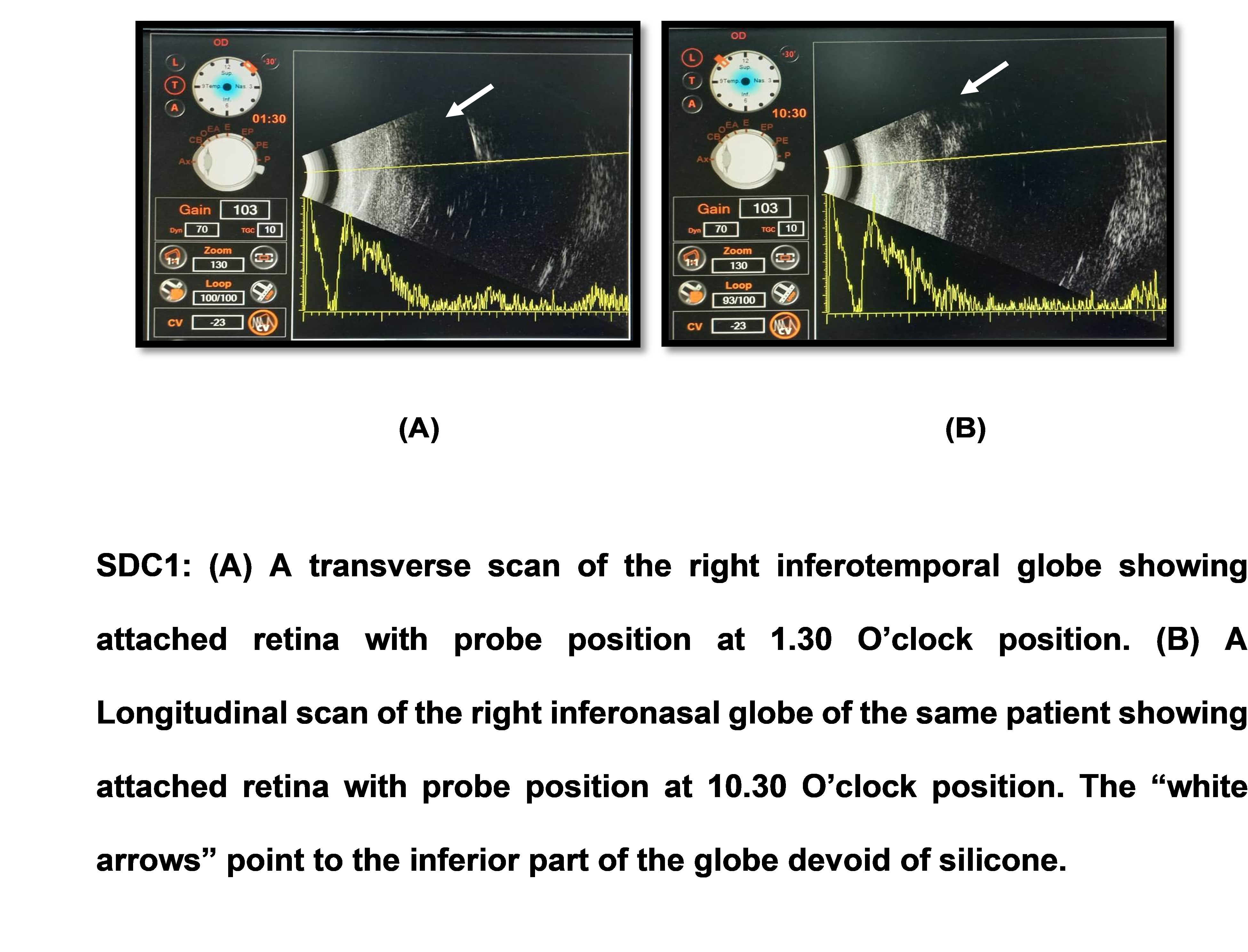

Supplement: Supplementary file 5 — SDC 1 [file 41433_2023_2464_MOESM5_ESM.tif]

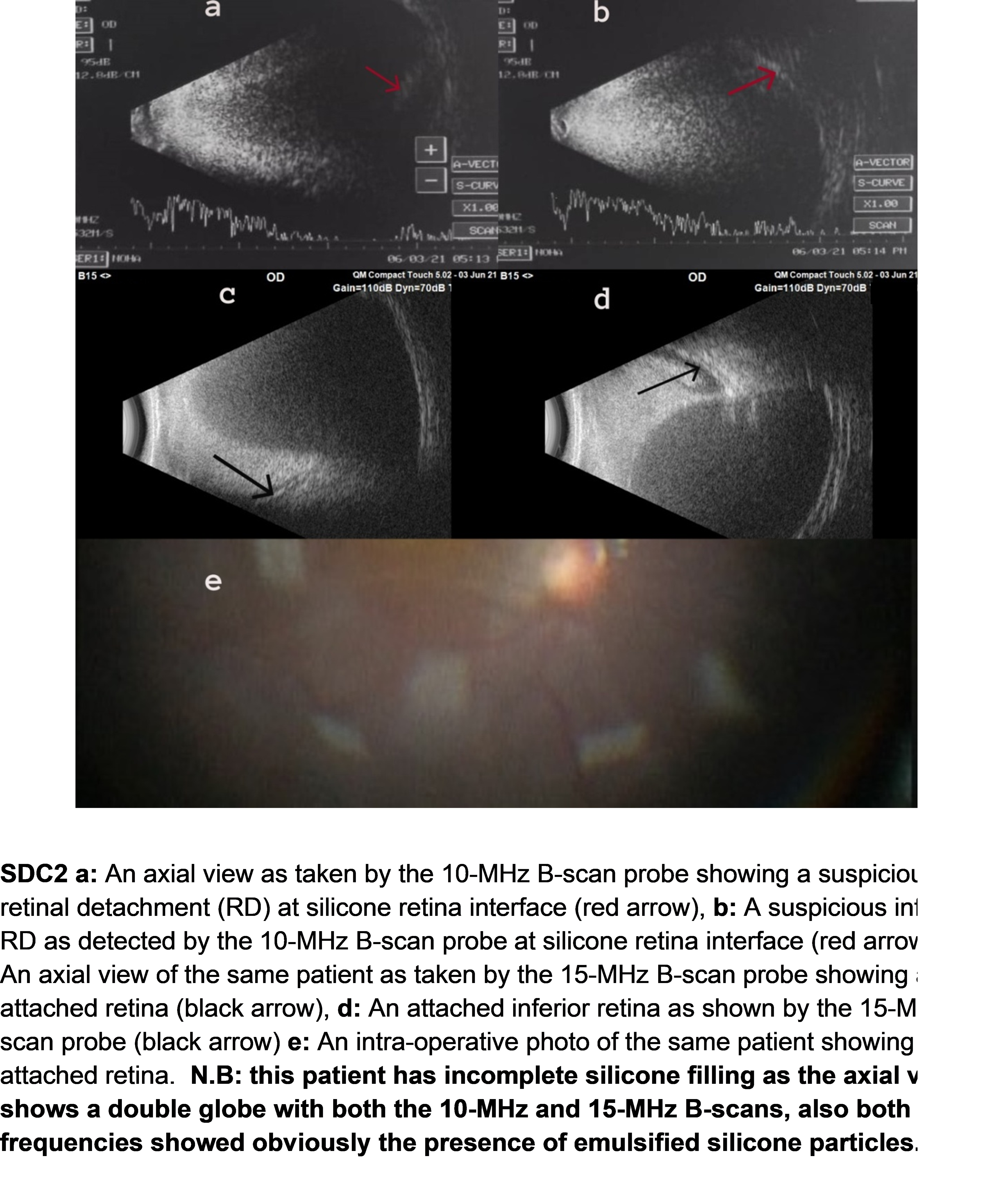

Supplement: Supplementary file 6 — SDC 2 [file 41433_2023_2464_MOESM6_ESM.tif]
