# Supplementary material for: A comparative study between 10-MHz and 15-MHz ultrasound probes for retinal evaluation in silicone-oil-filled globes
Source: Eye (Lond). 2023 Mar 6;37(14):3020–5. doi: 10.1038/s41433-023-02464-5 (PMC10516990; doi:10.1038/s41433-023-02464-5)
Supplement: Supplementary file 9 — Supplemental information [file 41433_2023_2464_MOESM9_ESM.docx]

Supplemental information 1: (A) A transverse scan of the right inferotemporal globe showing attached retina with probe position at 1.30 O’clock position. (B) A Longitudinal scan of the right inferonasal globe of the same patient showing attached retina with probe position at 10.30 O’clock position. The “white arrows” point to the inferior part of the globe devoid of silicone.

Supplemental information 2: a: An axial view as taken by the 10-MHz B-scan probe showing a suspicious retinal detachment (RD) at silicone retina interface (red arrow), b: A suspicious inferior RD as detected by the 10-MHz B-scan probe at silicone retina interface (red arrow), c: An axial view of the same patient as taken by the 15-MHz B-scan probe showing an attached retina (black arrow), d: An attached inferior retina as shown by the 15-MHz B-scan probe (black arrow) e: An intra-operative photo of the same patient showing an attached retina. N.B: this patient has incomplete silicone filling as the axial view shows a double globe with both the 10-MHz and 15-MHz B-scans, also both frequencies showed obviously the presence of emulsified silicone particles.

Supplemental information 3: A video illustration of the 15-MHz ultrasonography scan for a patient having an inferonasal retinal detachment under silicone.

Supplemental information 4: A video illustration of the 10-MHz ultrasonography scan for a patient having an inferonasal retinal detachment under silicone (the same patient illustrated in Supplemental information 3).
